# Comparison of deep learning networks for fully automated head and neck tumor delineation on multi-centric PET/CT images

**DOI:** 10.1186/s13014-023-02388-0

**Published:** 2024-01-08

**Authors:** Yiling Wang, Elia Lombardo, Lili Huang, Michele Avanzo, Giuseppe Fanetti, Giovanni Franchin, Sebastian Zschaeck, Julian Weingärtner, Claus Belka, Marco Riboldi, Christopher Kurz, Guillaume Landry

**Affiliations:** 1 Department of Radiation Oncology, LMU University Hospital, LMU Munich, Munich, Germany; 2 Department of Radiation Oncology, Radiation Oncology Key Laboratory of Sichuan Province, Sichuan Clinical Research Center for Cancer, Sichuan Cancer Hospital & Institute, Sichuan Cancer Center, Affiliated Cancer Hospital of University of Electronic Science and Technology of China, Chengdu, China; 3 Centro di Riferimento Oncologico di Aviano (CRO) IRCCS, Medical Physics, Aviano, Italy; 4 Centro di Riferimento Oncologico di Aviano (CRO) IRCCS, Radiation Oncology, Aviano, Italy; 5 Radiation Oncology, Charité-Universitätsmedizin Berlin, Freie Universität Berlin, Berlin, Germany; 6 German Cancer Consortium (DKTK), Partner Site Munich, Munich, Germany; 7 Bavarian Cancer Research Center (BZKF), Munich, Germany; 8 Department of Medical Physics, Ludwig-Maximilians-Universität München, Garching, Germany

**Keywords:** Head and Neck cancer, PET/CT, Tumor localization, Auto-segmentation, Facility-specific transfer learning

## Abstract

**Objectives:**

Deep learning-based auto-segmentation of head and neck cancer (HNC) tumors is expected to have better reproducibility than manual delineation. Positron emission tomography (PET) and computed tomography (CT) are commonly used in tumor segmentation. However, current methods still face challenges in handling whole-body scans where a manual selection of a bounding box may be required. Moreover, different institutions might still apply different guidelines for tumor delineation. This study aimed at exploring the auto-localization and segmentation of HNC tumors from entire PET/CT scans and investigating the transferability of trained baseline models to external real world cohorts.

**Methods:**

We employed 2D Retina Unet to find HNC tumors from whole-body PET/CT and utilized a regular Unet to segment the union of the tumor and involved lymph nodes. In comparison, 2D/3D Retina Unets were also implemented to localize and segment the same target in an end-to-end manner. The segmentation performance was evaluated via Dice similarity coefficient (DSC) and Hausdorff distance 95th percentile (HD_95_). Delineated PET/CT scans from the HECKTOR challenge were used to train the baseline models by 5-fold cross-validation. Another 271 delineated PET/CTs from three different institutions (MAASTRO, CRO, BERLIN) were used for external testing. Finally, facility-specific transfer learning was applied to investigate the improvement of segmentation performance against baseline models.

**Results:**

Encouraging localization results were observed, achieving a maximum omnidirectional tumor center difference lower than 6.8 cm for external testing. The three baseline models yielded similar averaged cross-validation (CV) results with a DSC in a range of 0.71–0.75, while the averaged CV HD_95_ was 8.6, 10.7 and 9.8 mm for the regular Unet, 2D and 3D Retina Unets, respectively. More than a 10% drop in DSC and a 40% increase in HD_95_ were observed if the baseline models were tested on the three external cohorts directly. After the facility-specific training, an improvement in external testing was observed for all models. The regular Unet had the best DSC (0.70) for the MAASTRO cohort, and the best HD_95_ (7.8 and 7.9 mm) in the MAASTRO and CRO cohorts. The 2D Retina Unet had the best DSC (0.76 and 0.67) for the CRO and BERLIN cohorts, and the best HD_95_ (12.4 mm) for the BERLIN cohort.

**Conclusion:**

The regular Unet outperformed the other two baseline models in CV and most external testing cohorts. Facility-specific transfer learning can potentially improve HNC segmentation performance for individual institutions, where the 2D Retina Unets could achieve comparable or even better results than the regular Unet.

## Introduction

Head and neck cancer (HNC), which is the sixth most frequently occurring cancer worldwide [1], is conventionally treated with radiotherapy, or radiotherapy-based combined modalities (chemotherapy and surgery) [2]. For radiotherapy, the delineation and segmentation of the gross tumor volume (GTV) from quantitative medical images, including the gross primary tumor volume (GTVp) and the associated lymph nodes (GTVn) [3, 4], is required and any inaccuracy can cause undertreatment of tumors and unnecessary irradiations of normal tissues. Labor-intensive and time-consuming manual delineation of the GTV from medical images is still the most common practice in clinics. However, due to the complicated HNC anatomical environment and irregular tumoral morphologies, manual segmentation can be error-prone and may suffer from intra/inter-observer variabilities [5]. The accurate identification and segmentation of the GTV remain crucial and challenging for HNC treatment.

It is widely accepted that [18 F]fluorodeoxyglucose (FDG) positron emission tomography (PET) and computed tomography (CT), providing both anatomical and metabolic information about the tumor, are two standard medical imaging modalities used for GTV segmentation during HNC diagnosis and radiotherapy treatment planning stages [6]. Compared with CT, PET can detect hypoxia levels [7] and reflect the physiological changes related to tumor cellular metabolism, thus serving as a relevant complementary source of information for tumor localization. However, PET can suffer from low spatial resolution and limited signal-to-noise [8]. The various FDG dosages and scanner settings from different vendors and institutions can also lead to large variations in PET image intensity. Thus, the more consistent and high-resolution anatomical information from CT is still indispensable for HNC GTV segmentation.

The recent development of deep learning methods has enabled auto-segmentation of the GTV in HNC, a competitive alternative to avoid time-consuming and error-prone manual delineation, validated by several studies [5, 10, 11] and challenges [12, 13]. Auto-segmentation of medical images is currently dominated by the Unet deep-learning architecture [9] and its variants [5, 10–13]. By adaptively adjusting the network architecture, training scheme, data pre-processing, and data post-processing, the Unet-based approaches could achieve Dice similarity coefficient (DSC) [14] scores from 0.71 to 0.78 for GTVp [10, 11] and 0.70 to 0.74 for combined GTVp and GTVn [5] segmentation.

Although it is common to have whole-body PET/CT scans in clinics, a tumor bounding box was usually selected manually in previous studies due to memory limitations. Such manual selection process can lead to an increase of the processing time. Besides, inter- and intra-physician variability may still be present. A solution for auto-localization and segmentation of HNC GTVs from entire images is desirable. Furthermore, previous studies were mainly trained and tested on datasets generated under the same guideline where the GTVp and GTVn were inspected and adapted beforehand [13]. Since variations in GTV delineation could still exist between institutions, the transferability of a trained model for external testing requires investigation.

The overall goal of this study was to explore the possibility of auto-localization and segmentation of HNC GTVs from entire PET/CT images and to investigate the transferability of the trained models for external testing with potential variations in GTV delineation style. We first used Retina Unet, a deep learning network for tumor localization [20], to find HNC from whole-body PET/CT scans, and successively utilized a regular Unet [25] to segment the GTV. Additionally, we also employed two end-to-end models for direct tumor localization and segmentation with 2D/3D Retina Unets. The segmentation performance between different models was compared via the Dice similarity coefficient (DSC) and Hausdorff distance (HD). Furthermore, to investigate the transferability of trained models, the prediction performance was additionally tested with data from three independent facilities. Finally, transfer learning, which has been beneficial for prostate cancer segmentation [15, 26], has also been performed to investigate whether the trained baseline models can further be adapted to the segmentation style of each external institution and thus improve the prediction performance.

## Material and method

### Dataset

We trained and cross-validated baseline models with the dataset provided by HECKTOR 2022, where 524 histologically proven HNC patients were collected from 7 different cohorts [12, 13] and where segmentations were retrospectively harmonized. The ground truth segmentation was based on human annotations of GTVp and GTVn, which were manually delineated by an annotator and cross checked by another. Precise contouring guidelines were elaborated to ensure the unification of all annotations. We then collected another 275 patients from three different cohorts for external testing. The MAASTRO (Maastricht Radiation Oncology clinic, Netherlands) cohort was publicly retrieved from the Cancer Imaging Archive (TCIA) [16–18], while the CRO (Centro di Riferimento Oncologico Aviano, Italy) and BERLIN (Charité-Universitätsmedizin Berlin, corporate member of Freie Universität Berlin, Radiation Oncology, Berlin, Germany) cohorts were obtained via collaboration. All clinical data were anonymously obtained and processed under relevant ethics approvals and regulations. Informed consent was obtained from all patients. The baseline characteristics of the patients from the different cohorts are summarized Table 1, where the tumor stage was omitted in the HECKTOR cohort since they were not publicly provided.

All patients underwent radiotherapy and/or chemotherapy treatment, had FDG-PET as well as non-contrast-enhanced CT images, and had the GTV (including GTVp and GTVn) contoured as segmentation masks. The contours of the CRO cohort were adaptively checked and modified for a radiomics study, while the contours of BERLIN were directly exported from the clinical treatment planning systems (TPS). Attributed to labels 1 and 2 (background with label 0), the GTVp and GTVn were contoured separately in the HECKTOR training dataset and MAASTRO cohort, while they were labelled together as 1 in the CRO cohort. For the BERLIN cohort, 12.9% of the patients had GTVp and GTVn contoured and labelled separately, while the rest (87.1%) had only a single label. Therefore, to unify the differences, we labelled GTVp and GTVn together as 1 and background as 0 in this study. The PET intensities had already been converted to standardized uptake values and the segmentation masks had been aligned with the corresponding CT images. The number of patients who had PET/CT scans exceeding 100 cm along the superior-inferior (SI) direction was 57 for the training dataset, and 53 for the external testing datasets.

**Table 1:** Baseline characteristics of the patients from different cohorts in this study

Cohort Name	Cohort description	Patients number	Male/ Female (%)	Median age (years)	Overall stage I/II/III/IV	Median GTV Volume (cm^3^)	Patients with SI scans > 100 cm
HECKTOR	Contours retrospectively harmonized	524	82/18	61	--	22.8	57
MAASTRO	Contours delineated by one radiologist	74	84/16	62	14/5/14/41	15.0	0
CRO	Contours re-checked for radiomics study	108	63/37	57	20/21/10/57	24.8	53
BERLIN	Contours directly exported from clinical TPS	93	83/17	60	1/5/20/67	60.4	0

### Image preprocessing

For the HECKTOR training dataset, although the PET and CT were registered, the average size and spacing of the PET scans were 200 × 200 × 200 voxels and 4 × 4 × 3 mm^3^, while for CT they were 512 × 512 × 200 voxels and 0.98 × 0.98 × 3 mm^3^. Therefore, resampling for PET/CTs and their GTV segmentation masks to a 1 × 1 × 1 mm^3^ isotropic grid was implemented via linear and nearest-neighbor interpolations, respectively. For the other testing cohorts (MAASTRO, CRO, and BERLIN), the registration of PET and CT was first verified. If the PET and CT images were not properly registered, an in-house python script was applied to replicate the clinical rigid registration with the provided DICOM files before resampling to 1 × 1 × 1 mm^3^. Finally, for all cohorts, the resampled PET and CT were re-scaled using z-scores (subtraction of the mean and division by the standard deviation).

### Tumor localization

**Fig. 1:**
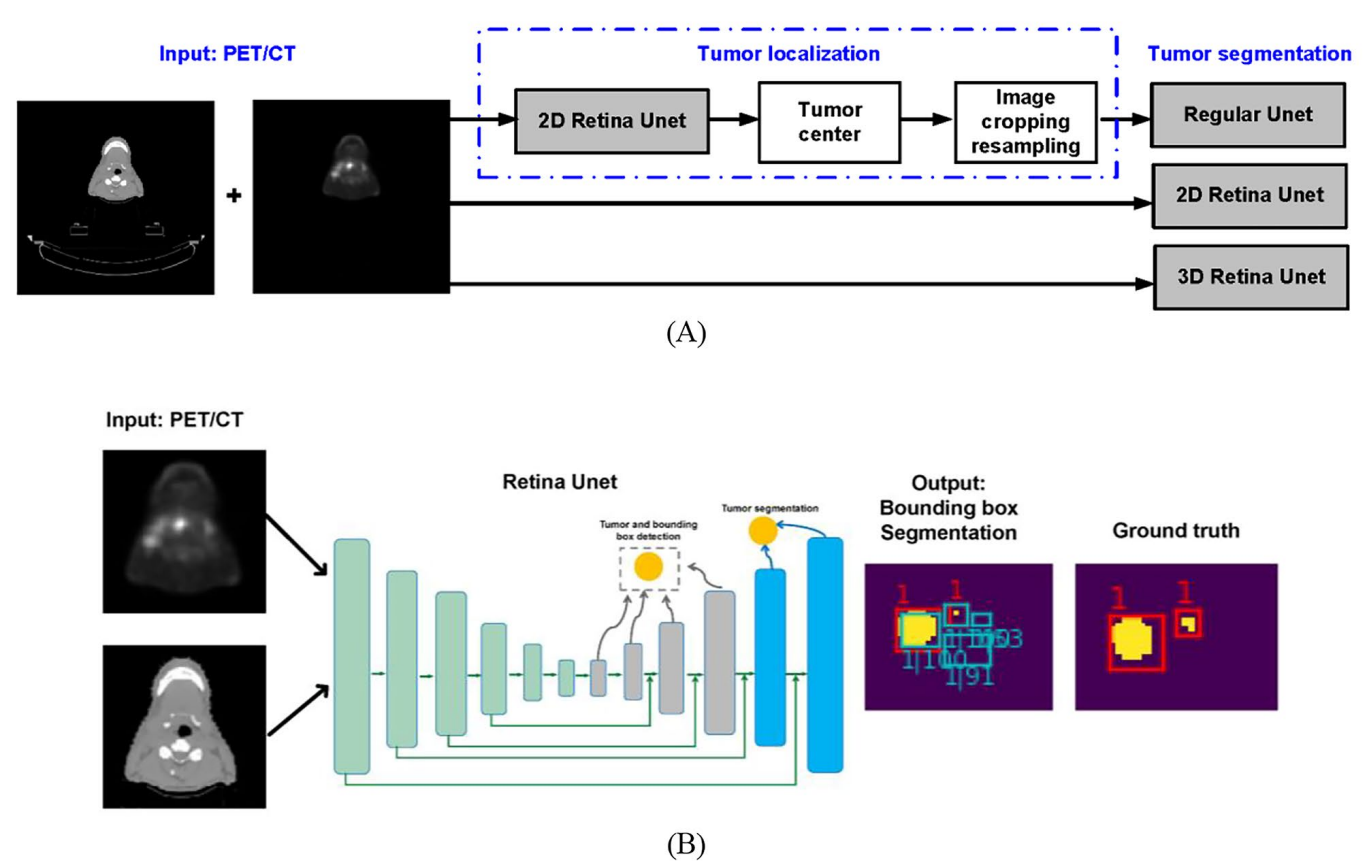
Illustration of the workflow for tumor localization and segmentation. **(A)** Segmentation schemes with regular Unet, 2D Retina Unet, and 3D Retina Unet. **(B)** Input and output of the Retina Unet. The green boxes denote the predicted bounding box and the confidence score are shown in white. The predicted segmentations are shown in yellow

We adopted the Retina Unet [20] in its 2D version to localize the tumor (combination of GTVp and GTVn) center from PET/CTs (including whole body scans). The dual-channel (with PET and CT as inputs) six-layer Retina Unet with ResNet50 [21] as the backbone was trained in a slice-based manner to determine the center of the tumor in each slice. Specifically, the Retina Unet outputs both the coordinates of a tumor bounding box and the corresponding confidence score (ranging from 0 to 1), as shown in Fig. 1(B). Since the GTVp and GTVn can be distinct volumes, there could be several predictions for each slice. To determine the center of the combined GTVp and GTVn, we computed the true and predicted bounding box center differences as a function of confidence score thresholds ranging from 0.4 to 0.9, effectively treating the threshold as a hyper-parameter optimized based on the validation set. If a bounding box had a confidence score larger than the threshold times the maximum confidence score of that patient, then it would be collected to compute the tumor volume center. Finally, we took the median center coordinates in 3D of those collected bounding boxes as the center for the tumor volume.

In our approach, the Retina Unet was trained with 80% of the HECKTOR dataset (419 patients) and validated on the remaining 20% (105 patients). A multi-task loss function was applied as

1 $${L_{retina\_unet}} = {L_c} + {L_b} + {L_s}$$

where *L*_*c*_ was the class loss defined as Eq. (5) in [22], *L*_*b*_ was the bounding box loss defined as Eq. (2) in [23], and *L*_*s*_ was the segmentation loss defined as a combination of soft Dice coefficient loss and the pixel-wise cross-entropy loss in Eq. (1) of [20]. To enhance the computational efficiency, the preprocessed PET/CTs and their segmentation masks were firstly cropped (from the axial image center) to a size of 512 × 512 mm in the axial plane with 2 mm pixel grid spacing and were resampled to 3 mm grid spacing along the superior-inferior direction, preserving the original superior-inferior length of the scans. For training, the input PET/CT images were randomly cropped into patches with a size of 128 × 128 pixels. Data augmentation was applied with a multithreaded augmentation pipeline [10], including scaling (from 0.8 to 1.1), rotation along the axial direction (from 0 to 360 degrees), and elastic deformation with parameter alpha in the range (0, 1500) and parameter sigma in the range (30, 50). The network was trained for 100 epochs using the Adam optimizer (learning rate 5e-4) on an NVIDIA Quadro RTX 8000 (48 GB) GPU with a batch size of 40. More details of the network architecture can be found in [20].

### Tumor segmentation

We used a dual channel 4-level Unet [25] to segment the GTV foreground (combination of GTVp and GTVn) as label 1. Based on the tumor center coordinates determined from the 2D Retina Unet, we first cropped and resampled the PET/CTs and their segmentation masks to a volume size of 256 × 256 × 256 mm on a 1 mm isotropic grid. With the default soft Dice loss function, the network was trained for 300 epochs using the Adam optimizer on an NVIDIA Quadro RTX A6000 (48 GB) GPU at a batch size of 4. The initial learning rate was 5e-4 and decayed at a rate of 2 if the validation loss was not improved after every 20 epochs. To avoid overfitting, the same pipline [10] for data augmentation was applied including a central random shift of maximal 16 voxels from the original image, a random mirroring with 50% probability, and an elastic deformation up to 25% of the cropped images.

In addition, since the Retina Unet [20] can localize the tumor center and simultaneously output the predicted foreground (as shown in Fig. 1(B)), we also implemented it in its 2D and 3D versions for tumor (combination of GTVp and GTVn) segmentation. For both Retina Unets, the same network structure and data augmentation process were applied. The loss function was kept the same as Eq. (1) with equal weightings for the multi-task losses. Besides, to focus more on the segmentation task, the weightings of L_*c*_, L_*b*_, and L_*s*_ on the right side of Eq. (1) were modified to be 0.1, 0.1, and 1.0, respectively. Besides, these two networks were trained for 200 epochs using the Adam optimizer (learning rate 1e-4) on an NVIDIA Quadro RTX 8000 (48 GB) GPU with a batch size of 40 and 8 for 2D and 3D cases, respectively. Similar to Sect. 2.3, the inputs of 2D Retina Unet were at a size of 256 × 256 pixels with a pixel spacing of 2 mm and were randomly cropped into patches with a size of 128 × 128 pixels during training. For 3D Retina Unet, the whole-body PET/CTs and their segmentation masks were resampled to a 2 × 2 × 3 mm grid after the preprocessing in Sect. 2.2. Later they were randomly cropped into patches with a size of 128 × 128 × 128 voxels during training.

The whole HECKTOR dataset was used for training and cross-validation (CV). For all three networks (regular Unet, 2D/3D Retina Unets), we applied 5-fold CV, resulting in five trained models with the highest average dice score for foreground (a combination of GTVp and GTVn). To get the segmentation masks for the testing datasets, the mean value of the predicted probability maps from all the CV folds was computed and thresholded at 0.5 to get the respective label masks. Figure 1(A) illustrated the segmentation schemes in this study.

### Adaptive filtering scheme for 2D/3D retina unets

To avoid segmentations from bounding boxes with low confidence scores, we implemented an adaptive filtering scheme. In this approach, thresholds with a range of 0.0 to 1.0 were evaluated for 2D/3D Retina Unets as hyper-parameters, which were optimized to improve the segmentation results during 5-fold cross-validation. If a bounding box had a confidence score larger than the threshold times the maximum confidence score of that patient, a binary mask corresponding to the box coordinates was constructed. This mask was then multiplied with the predicted segmentation of the patient, effectively removing predictions derived from bounding boxes with unsatisfactory confidence scores. Finally, all the multiplied segmentations were aggregated to build the filtered segmented predictions.

### Facility-specific training for segmentation networks

Due to the variability in GTV segmentation from independent facilities, the trained segmentation models (2D Retina, 3D Retina, Unet) from the HECKTOR dataset might not be transferable to the external testing datasets. Besides, it has been demonstrated in previous work that transfer learning from a baseline model could improve segmentation accuracy for independent institutions [26]. Therefore, we also extended the baseline segmentation model to three facility-specific models using transfer learning for the three external cohorts. The purpose of this approach was to adaptively adjust the baseline models to the different institutions. It should also be noted that the 2D Retina Unet for tumor localization was not re-trained with transfer learning. We only focused on the segmentation networks here.

For each segmentation network, the weights and biases were initialized with the baseline model and further trained and tuned with part of the PET/CTs randomly selected for each external facility. We implemented the transfer learning for each external cohort, and randomly selected 30% of the dataset for training and 20% for validation. The remaining 50% dataset of each external cohort was kept to test the models after facility-specific learning. Table 2 summarized the facility-specific training for the segmentation networks. The same data augmentation was employed to prevent overfitting, and the learning rate ranging from 1e-3 to 1e-6 was fine tuned. For 2D/3D Retina Unets, transfer learning was carried out with an NVIDIA Quadro RTX 8000 (48 GB) GPU. The transfer learning of the Unet was carried out on an NVIDIA Quadro RTX A6000 (48 GB) GPU.

**Table 2:** Number of training, validation, and testing patients for the facility-specific regular Unet, 2D Retina Unet and 3D Retina Unet

	Training	Validation	Testing
MAASTRO	22	15	37
CRO	32	22	54
BERLIN	28	18	47

### Evaluation metrics

To evaluate the segmentation performance, the predicted GTV contours were compared to their ground truth via Dice similarity coefficient (DSC) [14] and Hausdorff distance [27] at average (HD_avg_) and 95th percentile (HD_95_), respectively. In this study, we used Plastimatch [19] to compute DSC, HD_avg_ and HD_95_.

To verify if facility-specific training can significantly improve DSC and HD from the baseline models, the Wilcoxon signed-rank test was performed for the regular Unet and the 2D/3D Retina Unets, respectively. In addition, to compare the segmentation performances between different networks, we also implemented the non-parametric Friedman tests [28]. If the Friedman test revealed a significant difference (*p*-value < 0.05), a post-hoc Nemenyi test [29] was implemented to identify which network obtained significantly better DSC and HD in a pair-wise fashion.

## Results

### Tumor localization

**Fig. 2:**
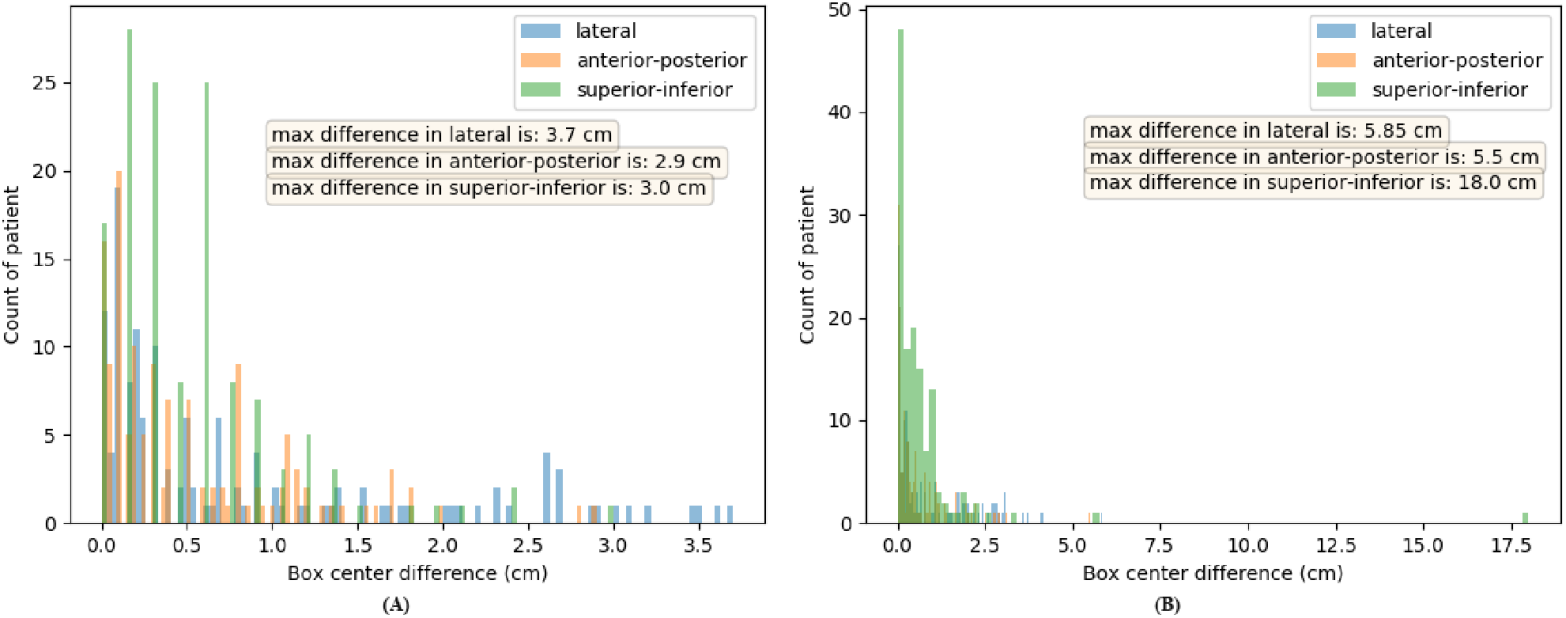
Histogram of tumor center differences (in cm) with thresholds **(A)** 0.6 and **(B)** 0.9. The values in lateral, anterior-posterior and superior-inferior directions are shown in blue, orange and green, respectively. The x-axis denotes the bounding box center difference in cm, and the y-axis denotes the patients count for the validation cohort (105 patients)

The maximum tumor center differences for the HECKTOR dataset are summarized in Table 3, where the confidence score threshold ranged from 0.4 to 0.9. For the training dataset, the differences were almost at the same level over different thresholds. Comparatively, the optimal threshold was 0.6 for the validation dataset, where the differences were always smaller than 4 cm in superior-inferior, lateral, and anterior-posterior directions, respectively [24]. Figure 2 displays the histogram of tumor center differences for the validation dataset at a confidence score threshold of 0.6 and 0.9, showing a higher threshold could lead to precise localization for most patients but might suffer from outliers. Therefore, to avoid this drawback, we chose the confidence score threshold as 0.6 in this study, accepting less accurate but more robust localization.

**Table 3:** Maximum tumor center differences in superior-inferior, lateral and anterior-posterior directions for the HECKTOR dataset (training and validation)

Threshold	Training dataset			Validation dataset		
	Superior-inferior	Lateral	Anterior-posterior	Superior-inferior	Lateral	Anterior-posterior
	(cm)					
0.4	6.5	4.6	3.2	3.3	5.4	2.9
0.5	6.0	4.6	3.2	3.2	5.7	2.9
0.6	6.0	4.7	3.1	3.0	3.7	2.9
0.7	5.6	4.7	3.2	18.0	4.0	2.9
0.8	5.5	4.9	4.1	18.0	5.8	2.9
0.9	5.4	4.9	3.3	18.0	5.8	5.5

**Fig. 3:**
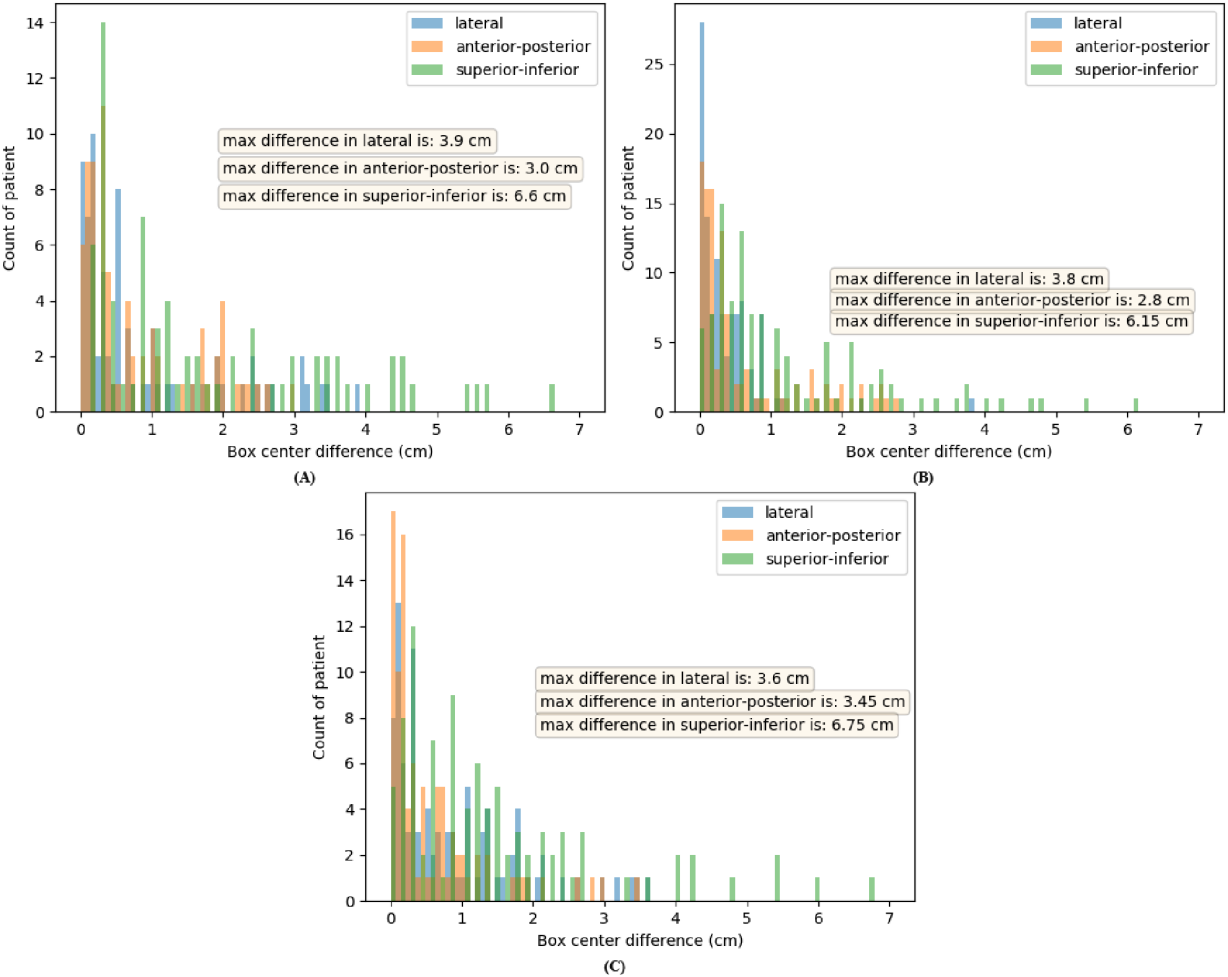
Histogram of tumor center differences (in cm) with a threshold of 0.6 for external testing cohorts. **(A)** MAASTRO (70 patients), **(B)** CRO (108 patients) and **(C)** BERLIN (93 patients)

The maximum tumor center difference for the external testing cohorts was also computed. With confidence score thresholds of 0.6, the tumor center differences in lateral, anterior-posterior and superior-inferior directions were (3.9, 3.0, 6.6), (3.8, 2.8, 6.2) and (3.6, 3.5, 6.8) cm for the MAASTRO, CRO, and BERLIN cohorts, respectively. The histogram of the tumor center differences is displayed in Fig. 3 without any outlier beyond 7 cm observed.

### Adaptive filtering for 2D/3D retina unets

For the 2D Retina Unet, various threshold values (0, 0.45, 0.65, 0.85, 0.90, 0.95, 0.99, 1.0) were examined. Compared with the model without filtering (threshold set to be 0), improvements in DSC were observed for all threshold values. As the threshold value increased from 0.45 to 0.90, the DSC consistently improved. The highest average cross-validation DSC of 0.71 was achieved and remained stable when the threshold values were set to be 0.90 and 0.95. When the threshold was 0.99, the DSC began to decline. Subsequently, the results were compared in terms of Hausdorff Distance at threshold values of 0.90 and 0.95. The averaged CV HD_avg_ / HD_95_ were found to be 3.2 / 11.3 mm for threshold 0.90 and 3.1 / 10.9 mm for threshold 0.95. Therefore, the optimal filtering threshold for 2D Retina Unet was selected to be 0.95 throughout the rest of the study.

**Table 2:** Median (25% − 75% percentile) DSC and HD from 5-fold CV via baseline models.

		Fold 1	Fold 2	Fold 3	Fold 4	Fold 5
Regular Unet	DSC	0.78 (0.64–0.82)	0.73 (0.63–0.80)	0.74 (0.63–0.81)	0.74 (0.65–0.81)	0.70 (0.59–0.81)
	HD_avg_ (mm)	2.4 (1.6-5.0)	2.6 (1.8–3.7)	2.8 (1.9–4.5)	2.7 (1.8–4.3)	2.7 (1.5–4.7)
	HD_95_ (mm)	8.7 (4.5–18.4)	8.9 (4.5–15.2)	8.8 (5.5–18.2)	9.1 (4.8–17.3)	7.0 (3.8–18.4)
2D Retina	DSC	0.71 (0.58–0.79)	0.70 (0.52–0.79)	0.75 (0.65–0.81)	0.68 (0.57–0.74)	0.72 (0.62–0.79)
	HD_avg_ (mm)	3.2 (2.1–5.2)	2.9 (1.9–6.3)	3.0 (1.9–4.8)	2.1 (2.4–5.6)	2.8 (2.0-5.6)
	HD_95_ (mm)	10.8 (5.8–22.9)	10.6 (5.5–22.8)	10.4 (5.1–23.6)	12.2 (6.7–25.3)	10.3 (5.9–20.0)
3D Retina	DSC	0.74 (0.62–0.83)	0.73 (0.58–0.81)	0.74 (0.61–0.81)	0.67 (0.54–0.76)	0.67 (0.56–0.76)
	HD_avg_ (mm)	2.3 (1.6–3.9)	2.7 (1.6–4.8)	2.8 (2.0-5.1)	3.5 (2.3–6.2)	3.2 (2.1–5.7)
	HD_95_ (mm)	7.0 (4.3–16.7)	8.5 (4.1–18.8)	8.6 (5.0-23.6)	12.8 (6.5–21.7)	12.0 (5.7–20.0)

For the 3D Retina Unet, various threshold values (0, 0.3,0.5,0.7,0.9,0.95) were also examined, and similar trends in DSC and HD performance were observed as in the 2D counterpart. The optimal threshold value turned out to be 0.70, yielding best averaged CV DSC of 0.71 and HD_avg_ / HD_95_ of 2.9 / 9.8 mm. Consequently, the threshold value for adaptive filtering of 3D Retina Unet was chosen to be 0.70 in this study.

### Segmentation by baseline models

The DSC was computed for the GTV foreground (combination of GTVp and GTVn). Compared with the equally weighted multi-task losses function in Eq. (1), the loss function with weightings (0.1, 0.1, 1.0) for (*L*_*c*_, *L*_*b*_, *L*_*s*_) could yield higher averaged CV DSC values in both 2D (0.69 vs. 0.71) and 3D Retina Unets (0.70 vs. 0.71); these latter weightings were therefore selected for this study. The CV results are summarized in Table 2. In general, the regular Unet achieved the best averaged DSC of 0.74 for 5-fold CV. In contrast, the 2D and 3D Retina Unets obtained slightly lower averaged DSCs of 0.71.

**Fig. 4:**
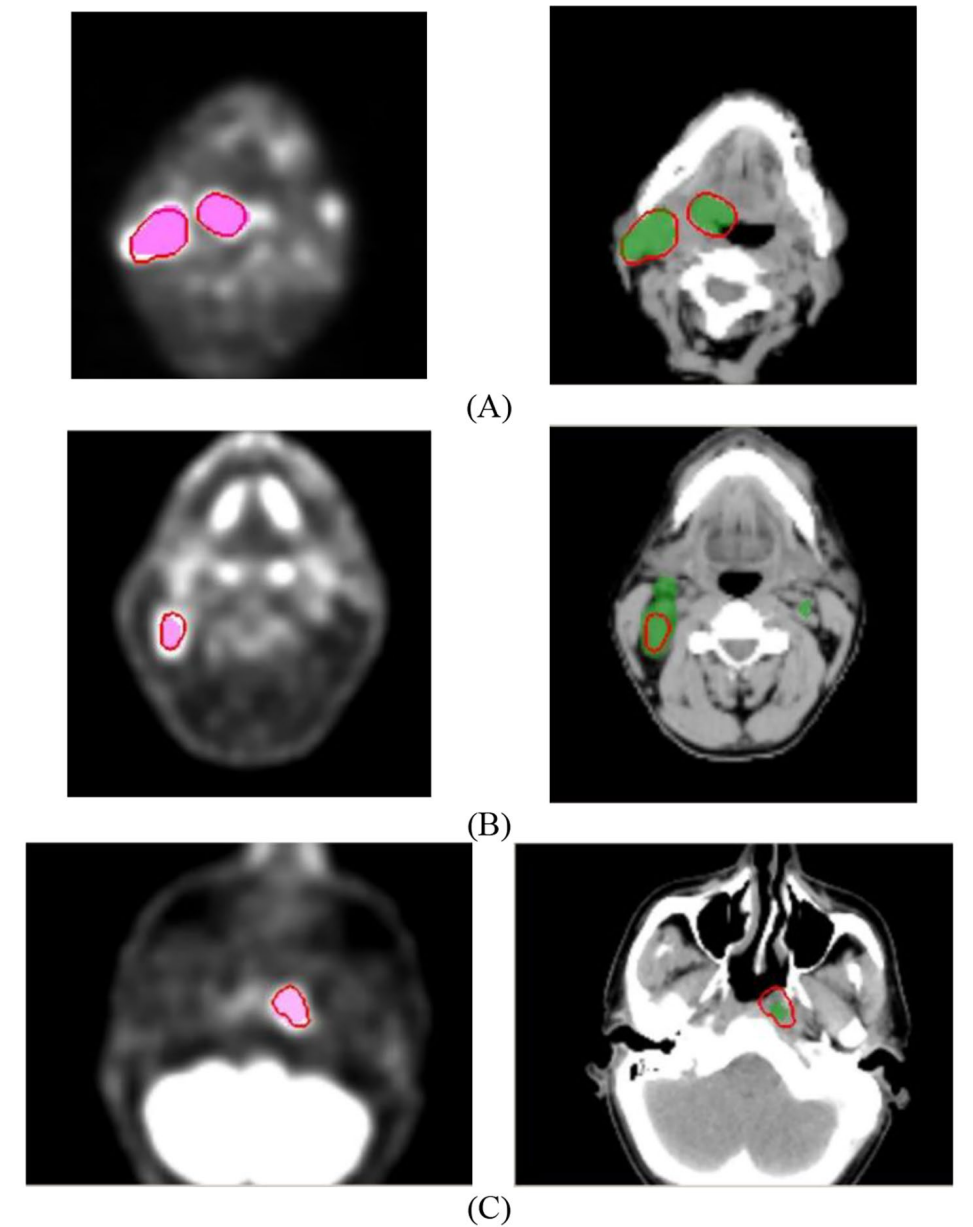
Image slices from the CRO cohort showing **(A)** one of the best, **(B)** average, and **(C)** poor baseline model performance. All the CTs are windowed with width of 200 and level of 20 in Housfield units (HU). The ground truth segmentation is contoured in red lines on both PET (left) and CT (right). The predicted GTV (combination of GTVp and GTVn) with the baseline regular Unet is displayed in green on CT.The predicted GTV from the facility-specific transfer learning for the regular Unet is displayed in pink on PET

However, if these baseline models were directly applied to the external testing cohorts, rather low DSC scores were obtained as summarized in Tables 3, 4 and 5. Compared with the 2D/3D Retina Unets, the regular Unet produced the highest DSC scores of 0.60, 0.63, 0.52 for MAASTRO, CRO, and BERLIN cohorts, respectively. Besides, we also noted that the 2D Retina Unet output higher averaged DSC than its 3D counterpart in the MAASTRO and CRO testing cohorts (0.60 vs. 0.57, 0.64 vs. 0.56). For the BERLIN cohort, the two Retina Unets produced similar median DSC scores, which were both smaller than 0.50. Fig. 4 collects several exemplary slices showing cases with one of the best (DSC 0.87), average (DSC 0.63), and poor (DSC 0.40) predicted segmentation from the CRO cohort with the regular Unet. It was observed that the predicted GTV segmentation was highly related to regions with higher SUV values in the PET image. However, in several cases, the high SUV region could still be outside of the GTV, leading to false positive predictions or larger GTV segmentation, as shown in Fig. 4(B). Conversely, the low SUV region could also contain GTVn, thus leading to false negative predictions.

We also evaluated the HD_avg_/HD_95_ for each CV and external testing cohort. The results are summarized in Tables 2, 3, 4 and 5. For cross validation, the regular Unet obtained the best averaged CV HD_avg_/HD_95_ at 2.6/8.5 mm, outperforming the 2D (3.1/10.9 mm) and 3D (2.9/9.8 mm) Retina Unets. For the external testing, the regular Unet outperformed the other networks in MAASTRO and CRO cohorts, with HD_avg_/HD_95_ at 3.9/13.4 and 3.6/12.4 mm, respectively. In the BERLIN cohort, the 2D Retina Unet obtained the optimal result of HD_avg_ at 6.8 mm, and the 3D Retina Unet obtained the optimal result of HD_95_ at 18.8 mm. A clear increase of HD_avg_ /HD_95_ was observed when applying baseline models directly to the external testing cohorts. With the regular Unet, the exemplary patients in Fig. 4 had HD_avg_ /HD_95_ at 1.9/5.3 mm, 9.9/30.5 mm, 6.0/14.8 mm for (A), (B) and (C), respectively.

**Table 3:** Median (25% − 75% percentile) DSC and HD for MAASTRO cohort via baseline and facility-specific models.

	Regular Unet	2D Retina Unet	3D Retina Unet
	DSC HD_avg_ (mm) HD_95_ (mm)	DSC HD_avg_ (mm) HD_95_ (mm)	DSC HD_avg_ (mm) HD_95_ (mm)
Baseline model	**0.62(0.52–0.71)**	0.60 (0.22–0.66)	0.57 (0.10–0.68)
	**3.9 (2.7–6.1)**	5.1 (2.8-19.68)	6.0 (2.9–20.6)
	**13.4 (6.8–20.0)**	18.0 (6.8–46.3)	20.3 (7.4–57.2)
Facility-specific model	**0.70 (0.56–0.75)**	0.68 (0.59–0.78)	0.66 (0.46–0.75)
	**2.8 (1.9–3.9)**	4.9 (2.3–8.1)	4.8 (2.4–12.2)
	**7.8 (4.7–12.7)**	17.8 (8.2–43.1)	16.5 (5.8–34.6)

**Table 4:** Median (25% − 75% percentile) DSC and HD for CRO cohort via baseline and facility-specific models

	Regular Unet	2D Retina Unet	3D Retina Unet
	DSC HD_avg_ (mm) HD_95_ (mm)	DSC HD_avg_ (mm) HD_95_ (mm)	DSC HD_avg_ (mm) HD_95_ (mm)
Baseline model	**0.67 (0.53–0.74)**	0.64 (0.50–0.74)	0.56 (0.18–0.66)
	**3.6 (2.6–5.8)**	4.8 (2.7–10.1)	5.6 (3.9–17.2)
	**12.4 (6.6–21.8)**	17.2 (7.6–33.7)	18.3 (9.8–43.2)
Facility-specific model	0.73 (0.62–0.79)	**0.76 (0.68–0.83)**	0.71 (0.64–0.76)
	**2.9 (1.9–4.7)**	3.3 (1.8–5.7)	4.5 (2.7-7.0)
	**7.2 (4.6–14.6)**	14.7 (4.8–25.8)	12.4 (6.7–32.7)

**Table 5:** Median (25% − 75% percentile) DSC and HD for BERLIN cohort via baseline and facility-specific training models

	Regular Unet	2D Retina Unet	3D Retina Unet
	DSC HD_avg_ (mm) HD_95_ (mm)	DSC HD_avg_ (mm) HD_95_ (mm)	DSC HD_avg_ (mm) HD_95_ (mm)
Baseline model	**0.52 (0.42–0.64)**	0.47 (0.32–0.65)	0.48 (0.18–0.61)
	7.2 (5.4–8.7)	**6.8 (4.4–14.3)**	7.0 (4.4–14.6)
	18.9 (11.4–28.6)	19.6 (12.6–40.3)	**18.8 (12.9–34.3)**
Facility-specific model	0.65 (0.51–0.73)	**0.67 (0.55–0.83)**	0.62 (0.44–0.71)
	6.0 (3.8–7.7)	**3.8 (2.3–8.9)**	6.4 (4.0-12.5)
	14.1 (8.9–24.3)	**12.4 (6.1–28.1)**	17.7 (11.3–49.0)

### Segmentation from facility-specific training models

**Fig. 5:**
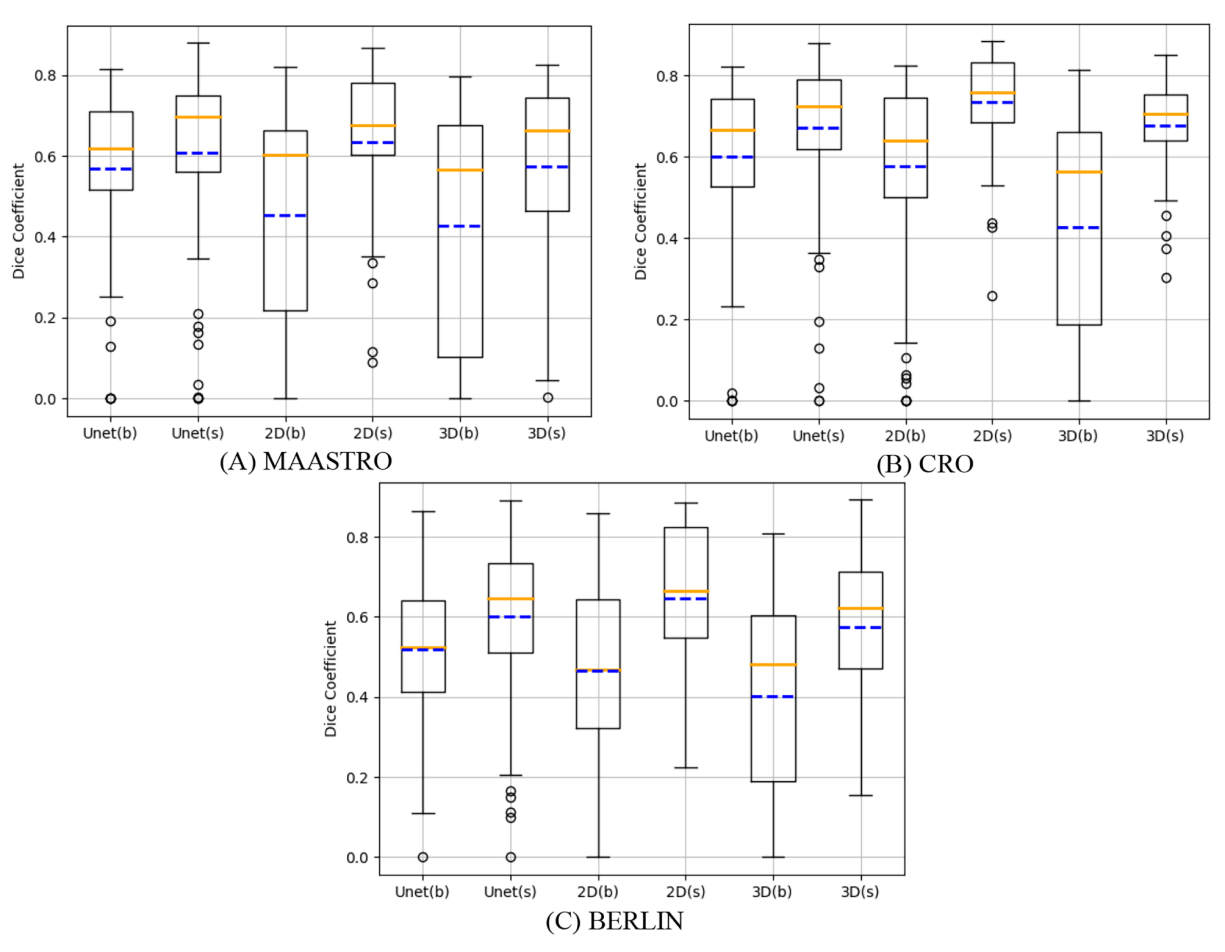
Box plots comparing the DSC of the baseline (b) and the facility-specific training (s) models, with solid orange line and dash blue lines representing the mean and median values of the DSC. Unet stands for regular Unet, 2D stands for 2D Retina Unet and 3D stands for 3D Retina Unet. **(A)** MAASTRO cohort. **(B)** CRO cohort. **(C)** BERLIN cohort

Tables 3, 4 and 5 also summarize the DSC and HD_avg_/HD_95_ results after facility-specific training for the three networks. The regular Unet produced the best segmentation results over others in the MAASTRO cohort with DSC of 0.70 and HD_avg_/HD_95_ of 2.8/7.8. For the CRO cohort, the regular Unet also achieved the best HD_avg_/HD_95_ of 2.9/7.2 mm, while the 2D Retina Unet achieved the best DSC result of 0.76. For the BERLIN cohort, it was still the 2D Retina Unet that produced the best segmentation results over the other two models with DSC of 0.67 and HD_avg_/HD_95_ of 3.8/12.4 mm. The DSC improvement after facility-specific training for each external cohort can also be seen in Fig. 5. Furthermore, according to the Wilcoxon signed-rank test between baseline models and facility-specific models in Table 6, significant improvements (*p* < 0.05) after the facility-specific transfer learning were observed for DSC, except from the regular Unet in the MAASTRO cohort. Besides, the HD_avg_ and HD_95_ were also significantly improved after facility-specific training in some external cohorts. In general, the facility-specific transfer learning could achieve enhancements in segmentation accuracy. With the facility-specific regular Unet, the DSC (HD_avg_/HD_95_) for the exemplary patients in Fig. 4 were 0.79 (2.5/5.5 mm), 0.71 (4.6/17.1 mm) and 0.58 (4.4/12.3 mm).

The Friedman test yielded significant differences among all the models in terms of DSC and HD_avg_/HD_95_. Therefore, a post-hoc Nemenyi test was applied, with results summarized in Table 7, to check which model obtained significantly better metrics in a pair-wise manner. For the DSC, the regular Unet showed significantly improved results compared to the 2D and 3D Retina Unets, while the 2D Retina Unet also showed significantly improved results against its 3D counterpart. For the HD_avg_, both the regular Unet and the 2D Retina Unet showed significantly improved results compared to the 3D Retina Unet. For the HD_95_, only the regular Unet performed significantly better than the 3D Retina Unet. There was no significant difference between the other models.

**Table 6:** *P*-value obtained from Wilcoxon signed-rank test between baseline models and facility-specific training models. Significant results (*p*<0.05) are denoted with an asterisk

	Regular Unet	2D Retina Unet	3D Retina Unet
	DSC HD_avg_ (mm) HD_95_ (mm)	DSC HD_avg_ (mm) HD_95_ (mm)	DSC HD_avg_ (mm) HD_95_ (mm)
MAASTRO	0.15	1.7e-3*	0.01*
	7.9e-4*	0.47	0.80
	4.8e-3*	0.96	0.69
CRO	3.7e-3*	< 1e-4*	< 1e-4*
	0.16	0.04*	0.13
	0.03*	0.21	0.39
BERLIN	0.048*	< 1e-4*	9.3e-4*
	0.11	4.1e-3*	0.74
	0.06	0.04*	0.22

**Table 7:** *P*-values obtained from the post-hoc Nemenyi test after facility-specific training for all possible pairwise model comparisons. Significant results (*p* < 0.05) are denoted with an asterisk

Comparison		DSC	HD_avg_	HD_95_
Model 1	Model 2	*p*-value		
Regular Unet	2D Retina Unet	0.04*	0.9	0.09
Regular Unet	3D Retina Unet	0.03*	1e-3*	1e-3*
2D Retina Unet	3D Retina Unet	1e-3*	1e-3*	0.06

## Discussion

In this study, several deep learning-based models were implemented to automatically localize and segment HNC tumors from entire PET/CT images. To avoid the manual selection of a bounding box, the 2D Retina Unet has been first used to localize the GTVs (combination of GTVp and GTVn) center, where the tumor center of each slice was selected using its confidence score. To find the optimal value of the confidence score threshold, we computed the tumor center differences with thresholds ranging from 0.4 to 0.9. According to the validation result in Table 3, the optimal threshold was 0.6, achieving a maximum difference of less than 6.8 cm in any direction in the three external testing cohorts. A higher value of 0.9 might have more precise localization results, but also suffered from outliers (maximum difference in superior-inferior direction was 18.0 cm). This might be caused by ignoring several lymph nodes with low confidence scores. Therefore, to compromise between precision and robustness, we chose 0.6 in this study.

Based on the localized tumor center, the PET/CT were cropped and input to a regular Unet for GTV segmentation. In comparison, we have also implemented two fully automated end-to-end models with 2D/3D Retina Unet, aiming to segment the HNC tumor directly from the whole-body PET/CT. The three baseline models were trained and cross-validated on the HECKTOR challenge dataset and later tested externally on MAASTRO, CRO, and BERLIN cohorts. For CV, the regular Unet outperformed the other models in terms of both DSC and HD, achieving averaged median DSC of 0.74 and HD_avg_ /HD_95_ of 2.6/8.5 mm, which were compatible with the published best result of combined GTVp and GTVn (DSC was around 0.74 and HD_95_ around 10 mm) where 153 patients collected from one institution were involved and a 3D Unet was used for training [5]. In their study, the GTVp and GTVn were firstly delineated with one oncologist and later reviewed and adapted with another radiologist and nuclear medicine physician [5]. Compared with the regular Unet, there was no clear difference in terms of DSC for the 2D/3D Retina Unets, while a maximum increase of 1.3/4.0 mm were observed in HD_avg_ /HD_95_.

However, a drop of more than 10% in DSC and a 40% increase in HD_95_ were observed if the baseline models were tested on the three external cohorts, suggesting models trained on the multicentric HECKTOR dataset might not be directly suitable for HNC tumor segmentation for other institutions. There might be variations in GTV delineation between the training and testing cohorts, which could potentially hamper the generalizability of the GTV segmentation. Although it was not specified which guidelines were used for GTV delineation, the MAASTRO and CRO cohorts were collected for radiomics studies with ground truth GTV segmentations checked and modified. Therefore, DSCs above 0.62 were obtained for these two cohorts with the best baseline model. In contrast, the GTV contours were directly exported from the TPS for the BERLIN cohort. Potentially, since no inspection has been done, the poorest segmentation results were observed with a DSC of merely 0.52.

When facility-specific training was applied, improvements in terms of DSC and HD_avg_/HD_95_ were obtained for all the baseline models. The regular Unet and the 2D Retina Unet were the best performing models, with best DSC (HD_avg_ /HD_95_) of 0.70 (2.8/7.8 mm) and 0.76 (2.9/7.2 mm) in MAASTRO and CRO cohorts, respectively. Even for the BERLIN cohort, the DSC (HD_avg_ /HD_95_) from the best model (2D Retina Unet) still achieved 0.67 (3.8/12.4 mm) after transfer learning, showing an increase of 42.5% in DSC and a decrease of 43.4% in HD_95_. The Wilcoxon signed-rank test again validated the enhancement of segmentation accuracy by transfer learning, suggesting the trained baseline model can still adapt to the individual segmentation style from the external institutions. Besides, according to the Nemenyi test, the regular Unet and the 2D Retina Unet yielded significantly higher DSC and lower HD_avg_ than the 3D Retina Unets after transfer learning. The smaller improvement in 3D Retina Unet could potentially suggest it might need more data during the transfer learning process.

Additionally, the adaptive filtering scheme could enhance the segmentation accuracy of both 2D and 3D Retina Unets. Retina Unets automatically generated bounding boxes and confidence scores during the segmentation. By applying spatial thresholding to the predicted GTVs, low-confidence predictions were effectively removed, resulting in improved segmentation performance.

Due to the retrospective nature of the study, there were uncertainties on the quality of the ground truth GTV in the external testing cohorts. We used the original datasets for the external testing and did not perform any additional inspection and adaptation of the delineations. Although the facility-specific training might partially adapt to the underlying differences between individual institutions, some pronounced variability could still impact performance. For datasets from multiple centers, the elaboration and adoption of a precise contouring guideline would be beneficial.

## Conclusion

In this study, three baseline models were trained to automatically localize and segment HNC GTVs on PET/CT images. The model using 2D Retina Unet and regular Unet for tumor localization and segmentation outperformed the other two end-to-end models with 2D/3D Retina Unets in the CV. This was also observed for most external testing cohorts, albeit with a low overall performance. Finally, the transferability of the baseline models was tested for three independent institutions, and encouraging testing results were observed after facility-specific training, where the optimal DSC and HD achieved by the regular or 2D Retina Unets were comparable with state-of-the-art studies.

## Data Availability

The HECKTOR and MAASTRO cohorts are publicly available datasets and can be found in https://hecktor.grand-challenge.org/Data/ and https://wiki.cancerimagingarchive.net/display/Public/Head-Neck-Radiomics-HN1. The CRO and BERLIN datasets are the result of collaborations and can be obtained upon request.

## Abbreviation

HNC: Head and neck cancer
GTV: Gross tumor volume
GTVp: Gross primary tumor volume
GTVn: The associated lymph nodes
CT: Computed tomography
PET: Positron emission tomography
DSC: Dice similarity coefficient
HD: Hausdorff distance
HD_95_: The 95th percentile of Hausdorff distance
HD_avg_: Average value of Hausdorff distance
CV: Cross-validation
FDG: Fluorodeoxyglucose
MAASTRO: Maastricht Radiation Oncology clinic, Netherlands
CRO: Centro di Riferimento Oncologico Aviano, Italy
BERLIN: Charité-Universitätsmedizin Berlin, corporate member of Freie Universität Berlin, Radiation Oncology, Berlin, Germany
*L*_*c*_: The class loss
*L*_*b*_: The bounding box loss
*L*_*s*_: The segmentation loss
